# Fertility Management After Tubal‐Preserving Surgery for a Mature Cystic Teratoma of the Fallopian Tube

**DOI:** 10.1155/crog/8827814

**Published:** 2026-07-22

**Authors:** Hiroaki Fujita, Aya Shiraiwa, Kana Hirayama, Minako Katano, Naoki Yoshikawa, Eri Nakano, Eri Obiya, Akane Takaya, Reiko Kitayama, Yoshihiro Nishijima, Yudai Tanaka

**Affiliations:** ^1^ Department of Obstetrics and Gynecology, Medical Park Shonan, Fujisawa, Kanagawa, Japan; ^2^ Department of Obstetrics and Gynecology, Medical Park Musashi-Kosugi, Kawasaki, Kanagawa, Japan

**Keywords:** hydrosalpinx, hysterosalpingography, in vitro fertilization, laparoscopy, mature cystic teratoma of the fallopian tube

## Abstract

Mature cystic teratoma of the fallopian tube is extremely rare, with no reports existing on infertility treatment following its surgical removal. We report a case of mature cystic teratoma identified within the right fallopian tube during laparoscopic surgery, with only the tumor resected while preserving the tube. Three‐month postoperative hysterosalpingography showed preserved tubal patency, but contrast agent retention in the pelvic cavity was noted during the delayed phase, suggesting peritubal adhesions. Subsequent timed intercourse failed to result in pregnancy, leading to in vitro fertilization. Laparoscopy prior to embryo transfer confirmed hydrosalpinx, prompting right salpingectomy. Clinical pregnancy was achieved following frozen‐thawed blastocyst transfer performed 2 months postoperatively. The pregnancy progressed well and resulted in delivery at term. Even when tubal preservation is chosen for mature cystic tubal teratoma, normal tubal function may not necessarily be maintained. Therefore, early postoperative hysterosalpingography and prompt laparoscopic evaluation with appropriate surgical intervention if abnormalities are detected are useful for improving subsequent infertility treatment outcomes.

## 1. Introduction

Mature cystic teratomas occurring in the fallopian tubes are extremely rare, with fewer than 100 cases reported. Due to the difficulty in distinguishing them from ovarian mature cystic teratomas on imaging, they are often discovered incidentally during surgery. Pathologically, they are benign tumors containing tissues from all three germ cell layers; however, as the possibility of malignancy cannot be ruled out preoperatively, many reports recommend laparoscopic fallopian tube resection as the treatment of choice [1–7].

In reproductive‐age women, the advantage of preserving the fallopian tube by removing only the tumor is that it leaves open the possibility of natural conception. However, there are no reports evaluating the outcomes of postoperative infertility treatment or pregnancy in women in whom the fallopian tube was preserved.

We report an extremely rare case of laparoscopic tumor resection with fallopian tube preservation in a patient with a mature cystic teratoma of the fallopian tube. In this case, after surgery, we performed a stepwise evaluation of tubal function using hysterosalpingography (HSG) and laparoscopy, followed by in vitro fertilization (IVF), which resulted in pregnancy, and she gave birth to a live baby. This patient′s course demonstrates that appropriate evaluation and treatment planning after tubal preservation are crucial for optimizing fertility prognosis.

## 2. Case Presentation

Three years prior to presentation, a 31‐year‐old woman (G0P0) was diagnosed as having a right ovarian cyst at another hospital. Two years ago, she underwent timed intercourse and intrauterine insemination at a local clinic in hopes of becoming pregnant but was unsuccessful and discontinued treatment 1 year ago. Because she was unable to conceive naturally, she visited our clinic hoping to resume infertility treatment. Transvaginal ultrasound and magnetic resonance imaging (MRI) revealed a 77‐mm cyst in the left adnexal region and two cystic lesions measuring 31 and 29 mm in the right adnexal region (Figure 1). Based on these findings, a bilateral mature cystic teratoma of the ovaries was initially suspected. Due to the large tumor size, laparoscopic surgery was performed prior to infertility treatment.

**Figure 1:**
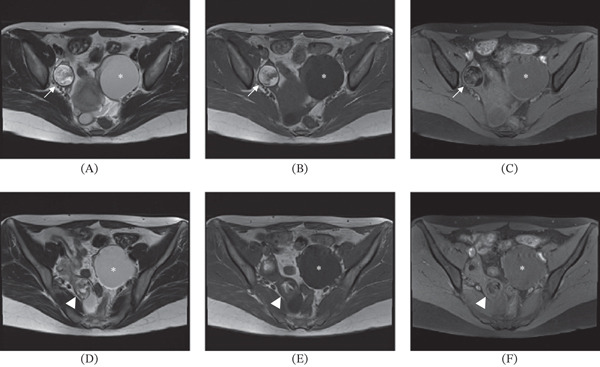
Magnetic resonance imaging of bilateral adnexal mature cystic teratomas. (A–C) A 31‐mm cystic tumor in the right ovary (→). (D–F) A 29‐mm tubular mass in the right fallopian tube (▲). Both lesions exhibit high signal intensity on T2‐weighted images (A and D) and on T1‐weighted images (B and E), whereas on fat‐suppressed T1‐weighted images (C and F), they contain fat with signal suppression, suggesting mature cystic teratomas. A 77‐mm cystic mass (*) is noted in the left ovary.

During surgery, mature cystic teratomas were found in both ovaries, and the right fallopian tube was dilated to 2 × 3 cm. Upon linear incision of the right fallopian tube, fat components and hair were found inside, suggesting a mature cystic teratoma. The left fallopian tube was normal. The patient had a strong desire to bear children. Furthermore, preoperative imaging did not suggest a fallopian tube‐related lesion, and consent for a salpingectomy had not been obtained from the patient. Therefore, after removing the teratomas from both ovaries, the right fallopian tube was preserved while removing the tumor and was sutured with 3‐0 absorbable suture (Polysorb; Covidien, Mansfield, Massachusetts) (Figure 2). An absorbable adhesion barrier (Interceed; Johnson and Johnson Medical Inc., New Brunswick, New Jersey) was also used. The surgery lasted 2 h and 18 min with blood loss of 100 g.

**Figure 2:**
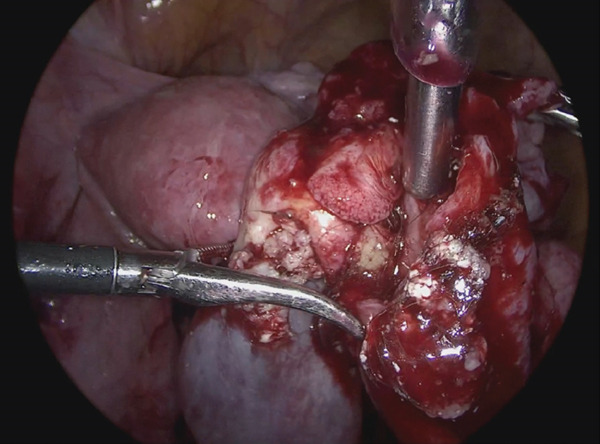
Laparoscopic removal of the mature cystic teratoma in the right fallopian tube. The cyst contained fat and hair.

Pathological examination of the right fallopian tube tumor revealed stratified squamous epithelial tissue with keratinization, sebaceous glands, hair follicles, hair, sweat glands, ciliated epithelium, and cartilage tissue, which led to a diagnosis of mature cystic teratoma. Postoperative reevaluation of the preoperative MRI revealed two separate lesions in the right adnexa. In retrospect, these could be identified as a cystic lesion originating from the ovary and a tubular lesion originating from the fallopian tube, respectively (Figure 1).

An HSG performed 3 months postoperatively showed that patency of both fallopian tubes was maintained, and contrast medium was observed flowing into the peritoneal cavity (Figure 3A). However, during the delayed phase 30 min later, contrast medium was retained in the pelvic cavity, suggesting peritubal adhesions (Figure 3B). Based on these findings, laparoscopic adhesiolysis was considered, but the patient expressed a desire to avoid invasive treatment, so surgery was not performed at this time. Subsequently, timed intercourse was performed over the next 6 months, but pregnancy did not occur. Therefore, IVF was initiated, and five blastocysts were cryopreserved after eight cycles of oocyte retrieval using controlled ovarian stimulation with clomiphene citrate and recombinant follicle‐stimulating hormone. Laparoscopic surgery was proposed to the patient prior to embryo transfer, and her consent was obtained.

**Figure 3:**
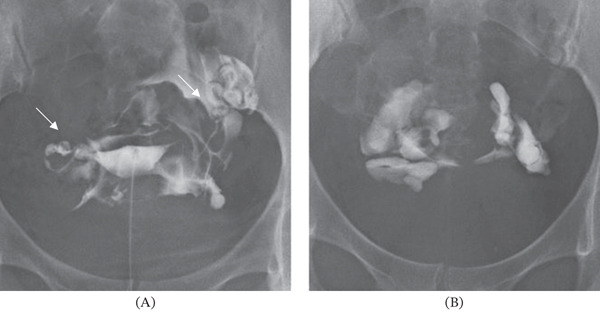
Postoperative hysterosalpingography (HSG). (A) Early‐phase HSG: Both fallopian tubes remain patent, and contrast medium is observed flowing into the peritoneal cavity (→). (B) Delayed‐phase HSG (30 min after contrast administration): Contrast agent is retained in the pelvic cavity, suggesting peritubal adhesions.

Intraoperative findings revealed marked dilation of the right fallopian tube ampulla, consistent with hydrosalpinx (Figure 4). As the hydrosalpinx was considered to interfere with embryo implantation after surgery, the right fallopian tube was therefore resected. The operation lasted 1 h and 3 min with minimal blood loss.

**Figure 4:**
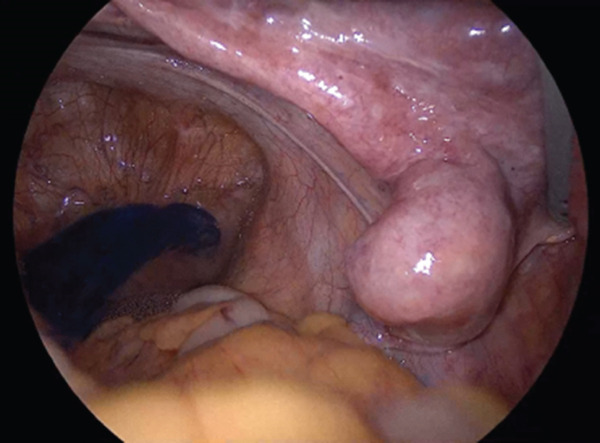
Right tubal hydrosalpinx confirmed by second‐look laparoscopy. The right ampulla is markedly dilated.

Macroscopic examination of the resected specimen revealed dilation of the fallopian tube lumen. Serous fluid had accumulated within the lumen, consistent with hydrosalpinx.

Two months postoperatively, a single cryopreserved‐thawed blastocyst (4AA) was transferred during a natural cycle. On day 10 posttransfer, her serum hCG was 419.3 mIU/mL, confirming pregnancy. The pregnancy progressed well, and due to breech presentation, delivery was performed via cesarean section at 38 weeks and 4 days.

## 3. Discussion

Mature cystic tubal teratomas are an extremely rare condition, most often diagnosed incidentally during surgery, with salpingectomy traditionally being the treatment of choice. However, there are no reports on the subsequent course of infertility treatment or pregnancy outcomes when tubal preservation is chosen in women of reproductive age. In this study, after choosing to preserve the fallopian tube for a mature cystic tubal teratoma, tubal function was evaluated stepwise during the course of infertility treatment. Based on these results, the treatment plan was revised, ultimately leading to pregnancy.

At 3 months postoperatively, HSG showed that fallopian tube patency was maintained, but the delayed phase revealed contrast agent retention in the pelvic cavity, suggesting peritubal adhesions. At that time, the pattern of contrast agent retention did not lead to an assumption of tubal hydrosalpinx, and tubal dysfunction due to adhesions was considered the primary cause of the failure of 6 months of timed intercourse. Adhesions around the fallopian tubes can impair egg pickup and transport within the fallopian tubes, even when tubal patency is maintained [8]. In a study of patients undergoing laparoscopic and hysteroscopic surgery for tubal factor infertility, the natural pregnancy rate was 72.1% for mild pelvic adhesions, 51.9% for moderate adhesions, and 27.9% for severe adhesions, indicating a significant decline as the severity of the adhesions increased [9].

During the laparoscopic examination performed prior to frozen embryo transfer, tubal hydrosalpinx was confirmed. Postoperative HSG alone is considered insufficient for adequately evaluating fallopian tube function. Recent studies suggest that hydrosalpinx reduces fertility through multiple mechanisms. Inflammatory cytokines and growth factors contained in the fluid accumulated in hydrosalpinx may adversely affect embryonic development and implantation; furthermore, the retrograde flow of this fluid into the uterine cavity may lead to mechanical expulsion of the embryo and a decrease in endometrial receptivity associated with chronic inflammation [10–14]. In addition, a previous meta‐analysis reported that clinical pregnancy and implantation rates following IVF are reduced by approximately 50% in patients with hydrosalpinx, indicating that hydrosalpinx adversely affects reproductive outcomes [15]. Furthermore, a recent meta‐analysis found that salpingectomy for hydrosalpinx significantly improved the rates of clinical pregnancy (OR 2.24, 95% CI 1.30–3.86) and ongoing pregnancy (OR 4.35, 95% CI 1.70–11.14) compared to no treatment [16]. In the present case, we considered that the tubal resection contributed to the subsequent early establishment of pregnancy.

Mature cystic teratomas of the fallopian tubes are extremely rare, and in many cases, it is difficult to distinguish them from ovarian tumors using imaging diagnosis [3–7]. The present patient was diagnosed preoperatively as having bilateral mature cystic teratomas of the ovaries; however, it was determined during surgery that one of the lesions in the right adnexa was a mature cystic teratoma originating from the fallopian tube. Had the presence of the fallopian tube‐derived lesion been diagnosed preoperatively, it might have been possible to propose a surgical procedure including fallopian tube resection during the initial surgery and plan the transition to IVF more efficiently. Improving the accuracy of preoperative MRI interpretation and establishing a diagnostic protocol that anticipates fallopian tube tumors are future challenges.

This report is of a single case at a single institution, and as the choice of treatment between tubal preservation or resection depends on the patient′s wishes and the institution′s experience, there are limitations to external validity. However, this report suggests that even after fallopian tube preservation, early HSG and additional evaluation via laparoscopy may contribute to optimizing subsequent infertility treatment. Although large‐scale studies are challenging due to the extreme rarity of mature cystic tubal teratomas, it is anticipated that accumulating further case reports will advance the consideration of diagnostic and therapeutic strategies.

As the present case shows, even if tubal preservation is chosen during treatment for mature cystic teratomas of the fallopian tubes, this does not necessarily mean that tubal function will be maintained normally after surgery. Therefore, we consider that performing HSG early after surgery and immediately conducting thorough examination and treatment by laparoscopy when abnormal findings are observed will contribute to the rapid identification of potential tubal lesions and improvement in infertility treatment outcomes.

## Funding

No funding was received for this manuscript.

## Consent

Written informed consent was obtained from the patient for publication of this case report and accompanying images.

## Conflicts of Interest

The authors declare no conflicts of interest.

## Data Availability Statement

Data sharing not applicable to this article as no datasets were generated or analyzed during the current study.
